# Palate anatomy and morphofunctional aspects of interpterygoid vacuities in temnospondyl cranial evolution

**DOI:** 10.1007/s00114-016-1402-z

**Published:** 2016-09-14

**Authors:** Stephan Lautenschlager, Florian Witzmann, Ingmar Werneburg

**Affiliations:** 1 School of Earth Sciences, University of Bristol, Bristol, BS8 1TQ UK; 2 Department of Ecology and Evolutionary Biology, Brown University, G-B204, Providence, RI 02912 USA; 3 Museum für Naturkunde, Leibniz-Institut für Evolutions- and Biodiversitätsforschung, Invalidenstraße 43, 10115 Berlin, Germany; 4 Senckenberg Center for Human Evolution and Palaeoenvironment (HEP) at Eberhard Karls Universität, Sigwartstraße 10, 72076 Tübingen, Germany; 5 Fachbereich Geowissenschaften der Eberhard Karls Universität Tübingen, Hölderlinstraße 12, 72074 Tübingen, Germany

**Keywords:** *Parotosuchus helgolandicus*, Biomechanics, Finite element analysis, Interpterygoid vacuities, Temnospondyli

## Abstract

Temnospondyls were the morphologically and taxonomically most diverse group of early tetrapods with a near-global distribution during the Palaeozoic and Mesozoic. Members of this group occupied a range of different habitats (aquatic, amphibious, terrestrial), reflected by large morphological disparity of the cranium throughout their evolutionary history. A diagnostic feature of temnospondyls is the presence of an open palate with large interpterygoid vacuities, in contrast to the closed palate of most other early tetrapods and their fish-like relatives. Although the function of the interpterygoid vacuities has been discussed in the past, no quantitative studies have been performed to assess their biomechanical significance. Here, we applied finite element analysis, to test the possibility that the interpterygoid vacuities served for stress distribution during contraction of the jaw closing musculature. Different original and theoretical skull models, in which the vacuities differed in size or were completely absent, were compared for their mechanical performance. Our results demonstrate that palatal morphology played a considerable role in cranial biomechanics of temnospondyls. The presence of large cranial vacuities were found to offer the dual benefit of providing additional muscle attachment areas and allowing for more effective force transmission and thus an increase in bite force without compromising cranial stability.

**Electronic supplementary material:**

The online version of this article (doi:10.1007/s00114-016-1402-z) contains supplementary material, which is available to authorized users.

## Introduction

Temnospondyls were the morphologically most diverse and species-rich group of early tetrapods with an evolutionary history that spanned a time interval of about 190 million years from the Early Carboniferous to the Early Cretaceous. Although different hypotheses have been discussed in the past, the group probably gave rise to modern lissamphibians, i.e., frogs, salamanders, and caecilians (Holmes 2000; Schoch 2014; for a different view of lissamphibian ancestry, see Marjanovic and Laurin 2013). Temnospondyls show a large spectrum of ecological adaptations to aquatic, semiaquatic, and terrestrial habitats and a great variety of body sizes, ranging from the newt- and salamander-like dissorophoids—as the potential lissamphibian ancestors—to the crocodile-like capitosaurian stereospondyls that reached up to 5 m body length (DeFauw 1989; Schoch and Milner 2000, 2014; Schoch 2014). A diagnostic feature of all temnospondyls is the presence of an open palate with large interpterygoid vacuities, in contrast to the closed palate of most other early tetrapods and tetrapodomorph fishes in which interpterygoid vacuities are either absent or small and slit-like (Laurin 2010; Clack 2012; Schoch 2014). Temnospondyls share the presence of large interpterygoid vacuities with extant frogs and salamanders, and assuming the derivation of lissamphibians from temnospondyls is correct, the open palate of both groups is a commonly derived character.

Although no study has been devoted so far to the functional significance of the interpterygoid vacuities in temnospondyls, three possible functions that need not to be mutually exclusive have been mentioned briefly in the literature: (I) the accessory function in buccal pumping of air into the lungs by raising and lowering of the skin that covered the interpterygoid vacuities by the levator and retractor muscles of the eye (Clack 1992; Laurin 2010; Schoch 2014); (II) the accessory function in swallowing by retraction of the eyeballs into the buccal cavity to push the prey toward the esophagus (Schoch 2014); (IIIa) the accommodation of an anterior portion of the jaw adductor musculature (Olson 1961) and (IIIb) force distribution during feeding. Hypothesis (I) was first formulated by Clack (1992) for the interpterygoid vacuities of temnospondyls, based on the presumed accessory role of the palate in air breathing of extant salamanders (Francis 1934), and this hypothesis was later adopted by Laurin (2010) and Schoch (2014). However, it is not clear if extant salamanders and frogs use this mode of breathing, and this has still to be tested experimentally. Hypothesis (II) is derived from observations in extant frogs and salamanders, in which the eyeballs indeed assist in swallowing prey (Deban and Wake 2000; Levine et al. 2004). It is very probable that this was also the case in temnospondyls, which show evidence for attachment sites of powerful, frog- and salamander-like retractor and levator eye muscles at the margins of the vacuities in temnospondyls (Witzmann and Werneburg 2016). However, this hypothesis does not explain the anterior extension of the interpterygoid vacuities far beyond the level of the orbits in several long-snouted temnospondyls, especially in capitosaurian stereospondyls. At this point, hypotheses (IIIa) and (IIIb) come into play. Olson (1961) was the first who mentioned that the interpterygoid vacuities of temnospondyls might have accommodated an anterior portion of the jaw adductor muscles, but he did not specify this further. We here concur with this view in the reconstruction of the cranial musculature in the capitosaur *Parotosuchus helgolandicus*, including an anterior portion of the jaw adductors that filled the middle and anterior part of the huge interpterygoid vacuities. The vacuities most likely served for the distribution of the tensile forces that occurred during contraction of the anterior muscle portion during feeding.

The aim of the present study is to test this latter hypothesis (interpterygoid vacuities as stress distribution of forces generated by an anterior adductor muscle) by using finite element analysis (FEA). We chose the skull of the capitosaur *P. helgolandicus* from the Early Triassic of Helgoland, Germany (Schroeder 1913; Schoch and Milner 2000), as a case study because this specimen shows an exceptional, uncrushed preservation and the elongate, huge interpterygoid vacuities as typical for capitosaurs.

## Material and methods

### Digitization and model generation

**Fig. 1:**
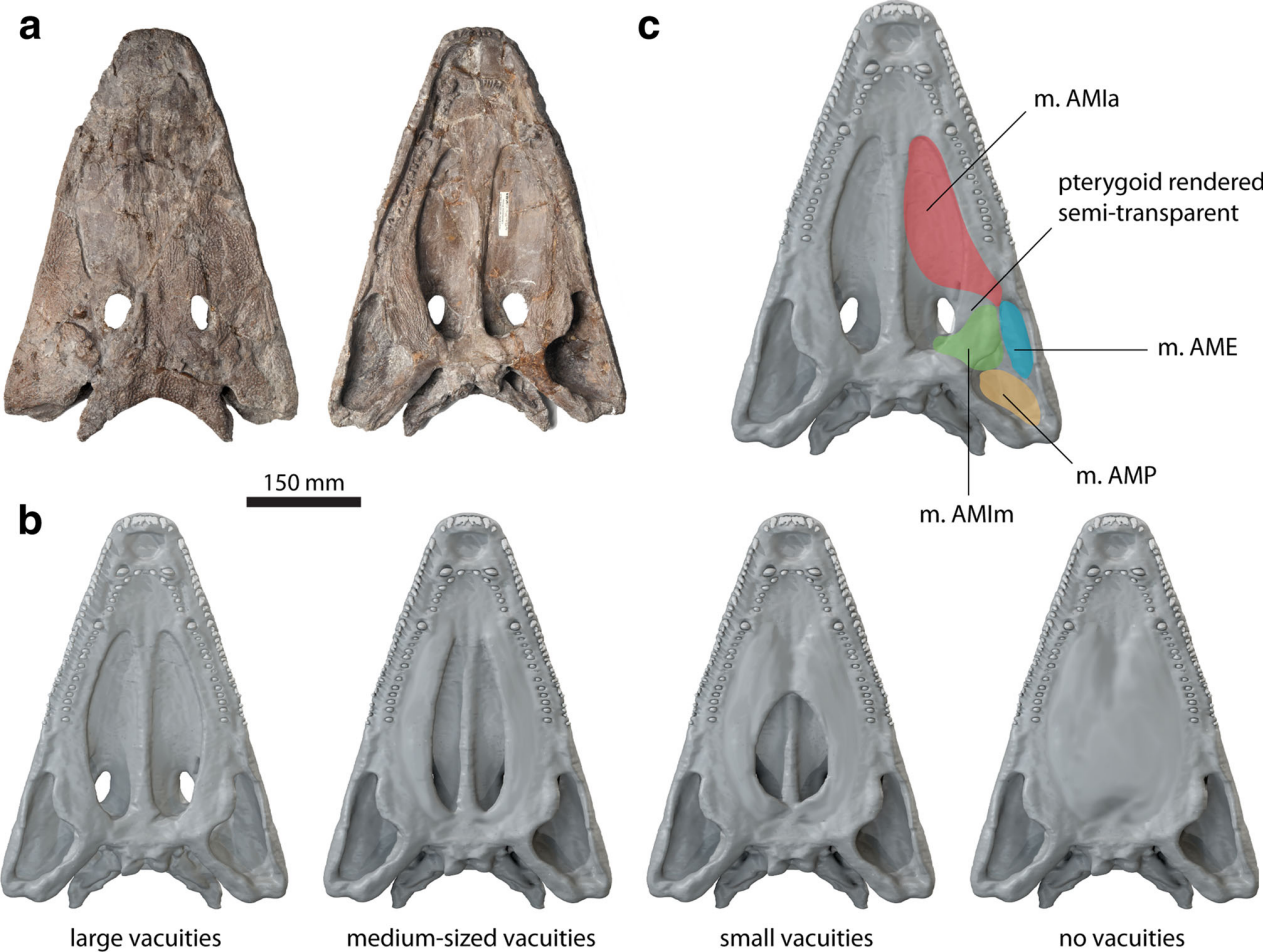
**a** Original specimen of *Parotosuchus helgolandicus* (MB.Am.841) in dorsal and ventral views. **b** Original and hypothetical models showing different configurations of the interpterygoid vacuities. **c** Cranial adductor muscle attachment sites mapped onto digital representation of specimen in ventral view

The complete and three-dimensionally preserved skull of the capitosaurian temnospondyl *P. helgolandicus* (MB.Am.841, Museum für Naturkunde, Berlin, Germany) from the Lower Triassic (lower Spathian) Middle Buntstandstein of Helgoland, Germany (Schroeder 1913; Schoch and Milner 2000) was selected as a template for the generation of digital models (Fig. 1a). The specimen was CT scanned at the Charité hospital Berlin, Germany, using an Aquilion ONE medical scanner with a resolution of 2 mm at 120 kV and 50 mA. Slice data consisting of 1781 slices with a slice thickness of 320 μm were imported into AVIZO (version 8.0; VSG, Visualization Science Group) for image segmentation and further processing. Moderate digital restoration was necessary to remove preservational artifacts, such as small breaks and cracks and to supplement some missing teeth. To correct for slight lateral distortion of the specimen, retrodeformation was performed with the geometric morphometrics software LANDMARK (version 1.6, www.idav.ucdavis.edu/research/EvoMorph; Wiley et al. 2005). Thirty pairs of bilaterally symmetric landmarks were selected on both sides of the specimen to calculate the plane of symmetry and to warp and symmetrize the model.

On the basis of the digitally restored model of *P. helgolandicus*, additional hypothetical models were created. These models incorporated different configurations of the interpterygoid vacuities representing distinct stages in early tetrapod cranial evolution and diversity of palatal morphology (Fig. 1b): (I) an original model representing the large interpterygoid vacuities of *P. helgolandicus*; (II) a model with medium-sized, more slit-like interpterygoid vacuities similar to the morphology found in the colosteid stem-tetrapod *Greererpeton burkemorani*; (III) a model with short and moderately wide interpterygoid vacuities similar to the morphology of *Cochleosaurus bohemicus* that belongs to the earliest lineages of temnospondyls, the Edopoidea (Schoch 2013), and its palatal morphology might therefore represent the plesiomorphic state for temnospondyls; and (IV) a completely hypothetical model with a fully closed interpterygoid region, e.g., similar to baphetid stem-tetrapods (Beaumont 1977) and—among lepospondyls—to forms like the nectridean *Batrachiderpeton* (Bossy and Milner 1998). For that purpose, hypothetical vacuity shapes were modeled in BLENDER (version 2.74, www.blender.org) using published skull reconstructions of *Greererpeton burkemorani* (Smithson 1982) and *Cochleosaurus bohemicus* (Sequeira 2003) as templates.

### Muscle attachments

For the calculation of muscle forces, the muscle attachments of the jaw adductor musculature of *P. helgolandicus* as reconstructed by Witzmann and Werneburg (2016) were considered (Fig. 1c). These authors, following the approach of Witzmann and Schoch (2013), used three steps for their reconstruction of the skull muscles. First, they took the well-preserved 3D architecture of the closed skull roof and the open palate of *Parotosuchus* as the basis to estimate the course and dimensions of the skull muscles. Second, structures on the bone surface like rugosities, crests, striations, depressions, or grooves were taken as osteological correlates of attachment sites for muscle fibers and tendons. In the third step, the extant phylogenetic bracket sensu Witmer (1995) was applied. In case a certain muscle (and its osteological correlate on a certain skull bone) was present in the bracketing taxa (e.g., dipnoans and anurans), then it could be reasonably assumed that a homologous muscle was probably also present in *P. helgolandicus*. According to the reconstruction of Witzmann and Werneburg (2016), the musculus (m.) adductor mandibulae posterior (m.AMP) originated on the internal (ventral) side of the squamosal and probably on its descending lamina as well as on the anterior part of the quadrate. M. adductor mandibulae externus (m.AME) originated on the internal side of the quadratojugal, the posteriormost part of the jugal, the lateral part of the squamosal, and possibly (to a lesser extent) on parts of the quadrate. It is hypothesized that m. adductor mandibulae internus (m.AMI) was divided into two parts, medial portion of m.AMI (m.AMIm) and anterior portion of m.AMI (m.AMIa). M.AMIm originated medially on the ventral side of the skull roof posterior to the orbit and on the neurocranium. M.AMIa is reconstructed as a long, preorbital head of m.AMI. Although there is no analogue of this muscle in extant amphibians, its presence was hypothesized in *P. helgolandicus* based on distinct attachment sites on the margins of the interpterygoid vacuities and the skull roof as well as the morphology of the adductor fossa (for details, see Witzmann and Werneburg (2016)). A hypothesized tendon of this muscle ran from the mandible dorsally into the anterior part of the adductor chamber of the skull and, for that, it was redirected around a ventral process of the jugal (the *insula jugalis*) in an anteromedial direction. Because of the extreme flattening of the skull in the preorbital region, the tendon is reconstructed as aligning at a very flat angle anterior to the *insula jugalis* and dorsal to the pterygoid. This tendon gave rise to a wide aponeurosis bearing the flattened AMIa that may have filled almost the complete interpterygoid vacuities anterior to the orbits and was dorsally directly attached to the skull roof, i.e., to parts of frontal, prefrontal, jugal, and lacrimal. Additionally, the aponeurosis was probably attached to the margins of the interpterygoid vacuities.

Muscle forces were calculated using the “dry skull method” (Thomason et al. 1990). For this purpose, the surface area of the cranial attachments was measured and multiplied by a specific tension value for vertebrate muscle (0.3 N/mm^2^) for the four adductor muscles (Table 1).

**Table 1:** Attachment locations and calculated muscle forces for cranial adductor musculature

Muscle	Location	Insertion area (mm^2^)	Force (N)
m.AME	Ventral surface of quadratojugal	8776	2632.8
m.AMIa	Ventral surfaces of frontal, prefrontal and jugal	2617	785.1
m.AMIm	Ventral surfaces of frontal, postfrontal and postorbital	2007	602.1
m.AMP	Ventral surface of squamosal	1658	497.4
Sum			4517.4

#### Finite element analysis

For the finite element analysis (FEA), all models were imported into HYPERMESH (version 11, Altair Engineering) for mesh cleaning and mesh generation. All models consisted of approximately 1.25 million four-noded tetrahedral elements. Previous studies on temnospondyl cranial biomechanics have used material properties for crocodilian cortical bone and teeth, suggesting that the microstructure (Witzmann et al. 2010) and heavy ornamentation of capitosaurs is functionally more similar to crocodilians than extant amphibians (Fortuny et al. 2011, 2012). Following this example, material properties obtained for extant crocodilians (Currey 1987; Creech 2004) were assigned in HYPERMESH (bone: *E* = 6.65 GPa, ν = 0.35; teeth: *E* = 60.40 GPa, ν = 0.31) and treated as homogenous and isotropic. All FE models were restrained from rigid body movement in all directions at the occipital condyle (ten nodes), the postparietal margin (seven nodes), and the quadrate (four nodes on each side). Further constraints were applied to different bite points according to the tested functional scenarios (see below).

To assess the biomechanical behavior of the modeled cranial configurations, different functional scenarios were tested: (i) unilateral biting at the left vomerine tusk (constrained at one node); (ii) bilateral biting at the vormerine tusks (constrained at one node each); (iii) lateral pull simulated by an extrinsic force of 200 N applied in lateral direction to the left vomerine tooth (this value was selected under the assumption that a neck-muscle-driven force will be in the same order of magnitude as the bite forces, but somewhat lower than the latter); (iv) unilateral biting at the middle of the left tooth row with constraints (one node) applied the palatine tusk; and (v) unilateral biting at the last tooth position of the left tooth row (constrained at one node). All configurations were analyzed with muscle forces applied to the ventral surface of the cranium. Additional analyses were performed with the same configurations, but with attachment of the m.AMIa extended onto the margins of the interpterygoid vacuities. Due to the lack of interpterygoid margins, this was not tested for the model with a fully closed palatal region. Instead, a sensitivity test was performed with additional attachments of the m.AMIa extending onto the dorsal surface of the closed palate. All models were subsequently imported into ABAQUS (version 6.14, Simulia) for analysis and postprocessing. Biomechanical performance was assessed by comparison of von Mises stress, deformation, and strain distribution and magnitude. In addition, reaction forces from the solved FE models at the bite points were recorded to obtain bite force measurements.

## Results

### Finite element analysis results

**Fig. 2:**
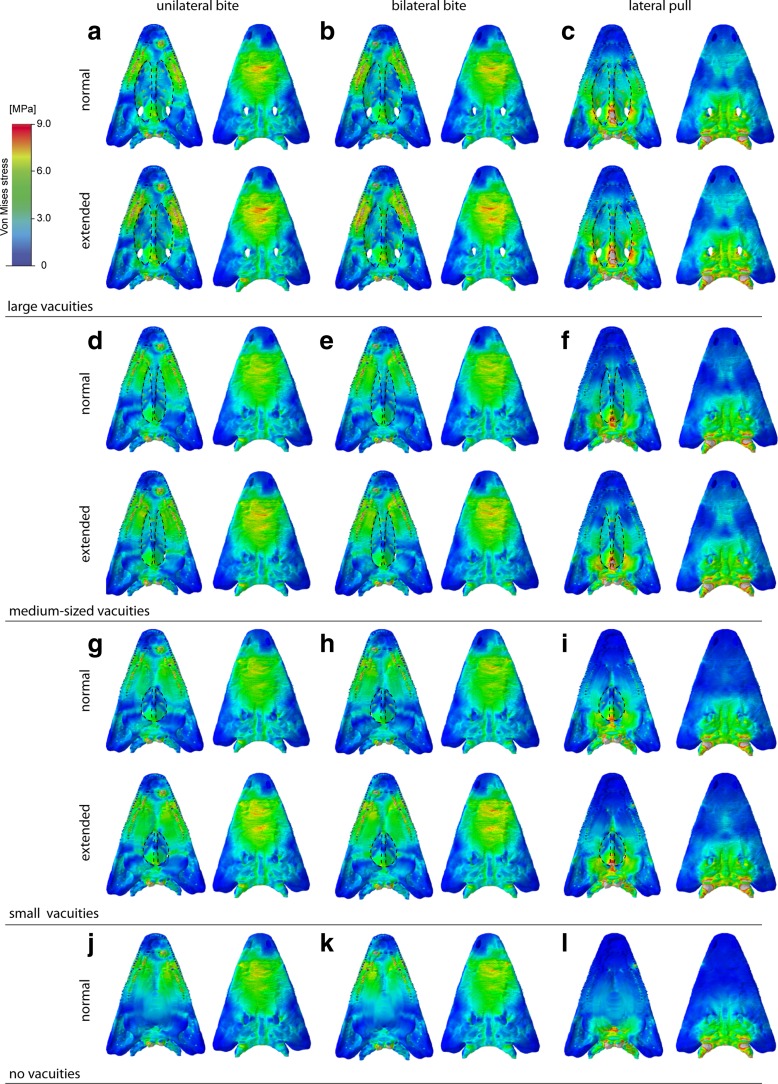
Von Mises stress contour plots for different tested cranial configurations: **a**–**c** original model, **d**–**f** medium-sized interpterygoid vacuities, **g**–**i** small interpterygoid vacuities, **j**–**l** closed palatal region. Different loading conditions: **a**, **d**, **g**, **j** unilateral bite on left side; **b**, **c**, **h**, **k** bilateral bite; **c**, **f**, **i**, **l** lateral pull to left side. Each in ventral and dorsal view. Location and size of the vacuities highlighted by *stippled line*

**Fig. 3:**
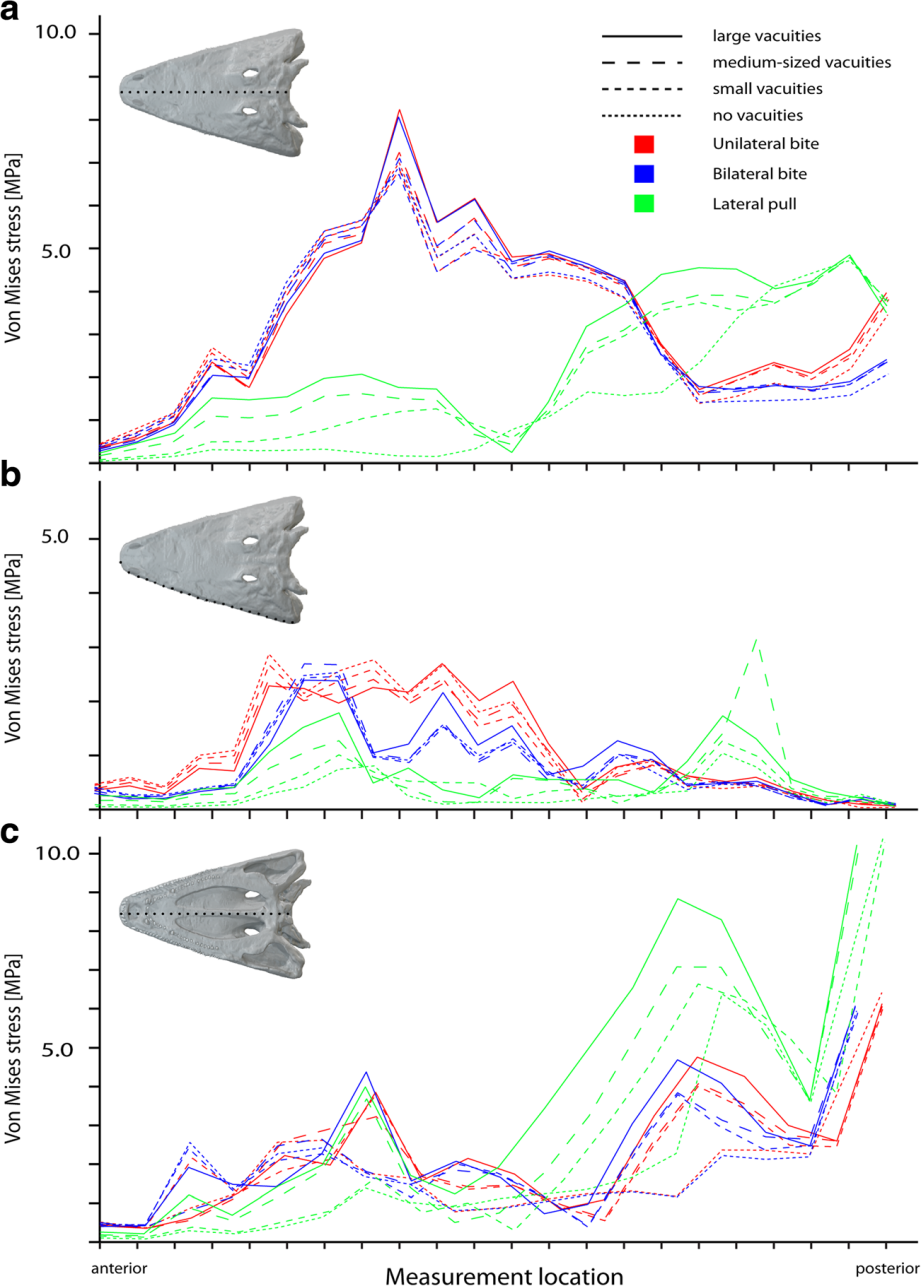
Von Mises stress values for different tested cranial configurations. Measured along **a** the skull roof, **b** the left lateral margin, **c** the ventral midline

We compared results of the finite element analyses for the different cranial configurations and conditions of the interpterygoid vacuities. All of them showed very similar patterns of von Mises stress distribution (Fig. 2). Maximum stresses were localized dorsally on the skull roof in the nasal and prefrontal region, and ventrally around the bite point on the maxilla and palatine. Similarly, stress magnitudes did not differ substantially for the measured nodes along the skull roof dorsally, the palatal region ventrally and along the lateral skull margin (Fig. 3) for the different configurations. The same distribution pattern was observed for maximum and minimum principal strain (Supplementary Figs. S1, S2, and S3). Modification of the interpterygoid vacuities by making them smaller or closing the vacuities completely resulted only in a minimal reduction of stress and strain magnitudes (Fig. 3). This effect was more pronounced for deformation magnitudes, which decreased moderately with the closing of the interpterygoid vacuities (Supplementary Fig. S7). The comparatively largest differences in von Mises stress magnitudes between the different cranial configurations were found for a lateral pull simulation. Here, stresses and strains decreased with the closing of the vacuities moderately along the ventral surface of the skull and most pronounced in the parasphenoid (Fig. 2, Supplementary Figs.S1. S2, and S3).

**Fig. 4:**
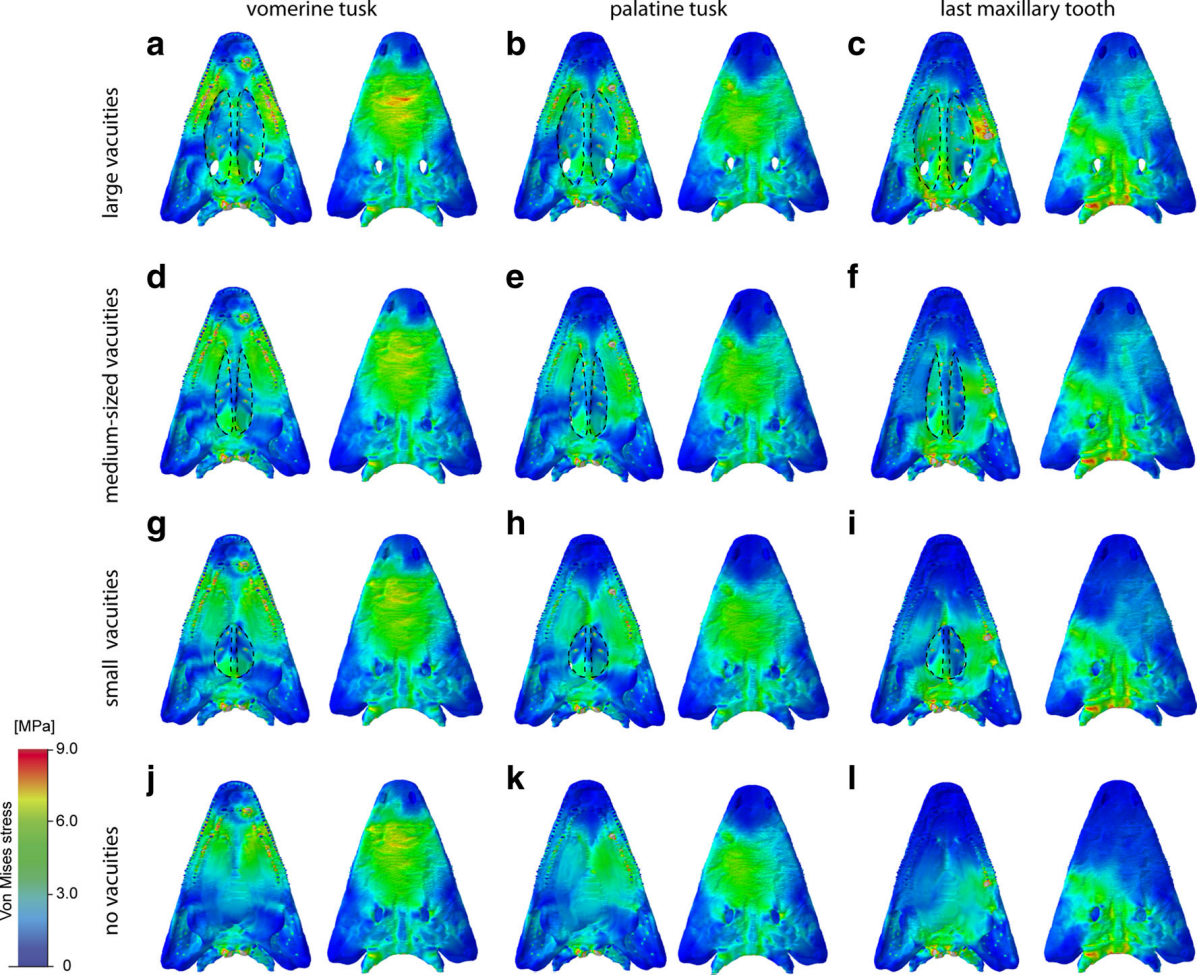
Von Mises stress contour plots for different tested cranial configurations and bite points: **a**–**c** original model, **d**–**f** medium-sized interpterygoid vacuities, **g**–**i** small interpterygoid vacuities, **j**–**l** closed palatal region. Different bite points: **a**, **d**, **g**, **j** vormerine tusk; **b**, **c**, **h**, **k** palatine tusk; **c**, **f**, **i**, **l** last maxillary tooth. Each in ventral and dorsal view. Location and size of the vacuities highlighted by *stippled line*

The analysis of different bite positions showed that there are no obvious differences in von Mises stress distribution and magnitudes between the simulation of unilateral and bilateral bites (Figs. 2 and 3). In comparison, moving the bite point from an anterior to middle and posterior positions along the tooth row resulted in a shift of stress hot spots from the nasal to the orbital region, the parietal, and the back of the skull (Fig. 4). Ventrally, a shift in bite position deflected the peak stresses from the maxilla to the pterygoid and the parasphenoid. This pattern was consistent also for maximum and minimum strains.

Von Mises stresses during a lateral pull differed greatly from the muscle-driven bite simulations (Fig. 2c, f, i, l). Highest stress magnitudes were found to center on the parietal region and the braincase, whereas the anterior skull region showed comparatively low stresses. The same pattern was recorded for maximum and minimum strains. In contrast, the peak deformation magnitudes were increased in the anterior part of the skull (Supplementary Figs. S4, S5, and S6).

The simulated extension of the m.AMIa from the ventral surface of the skull roof to the margins of the interpterygoid vacuities showed no differences in von Mises stress and strain distributions (Fig. 2, Supplementary Figs. S2 and S3). Only the stress and strain magnitudes were found to be slightly higher for all tested scenarios and configurations. As the closed interpterygoid vacuities provided no additional muscle attachments along the margins, only configurations with open vacuities were compared. However, to evaluate the effects on cranial stability, an extended attachment of the m.AMIa onto the dorsal surface of the closed palate similar to the condition in modern crocodilians was tested. Although this configuration changes the direction of the simulated muscle vectors, there were no notable differences observed for stress and strain distribution (Supplementary Fig. S10).

### Bite forces

**Fig. 5:**
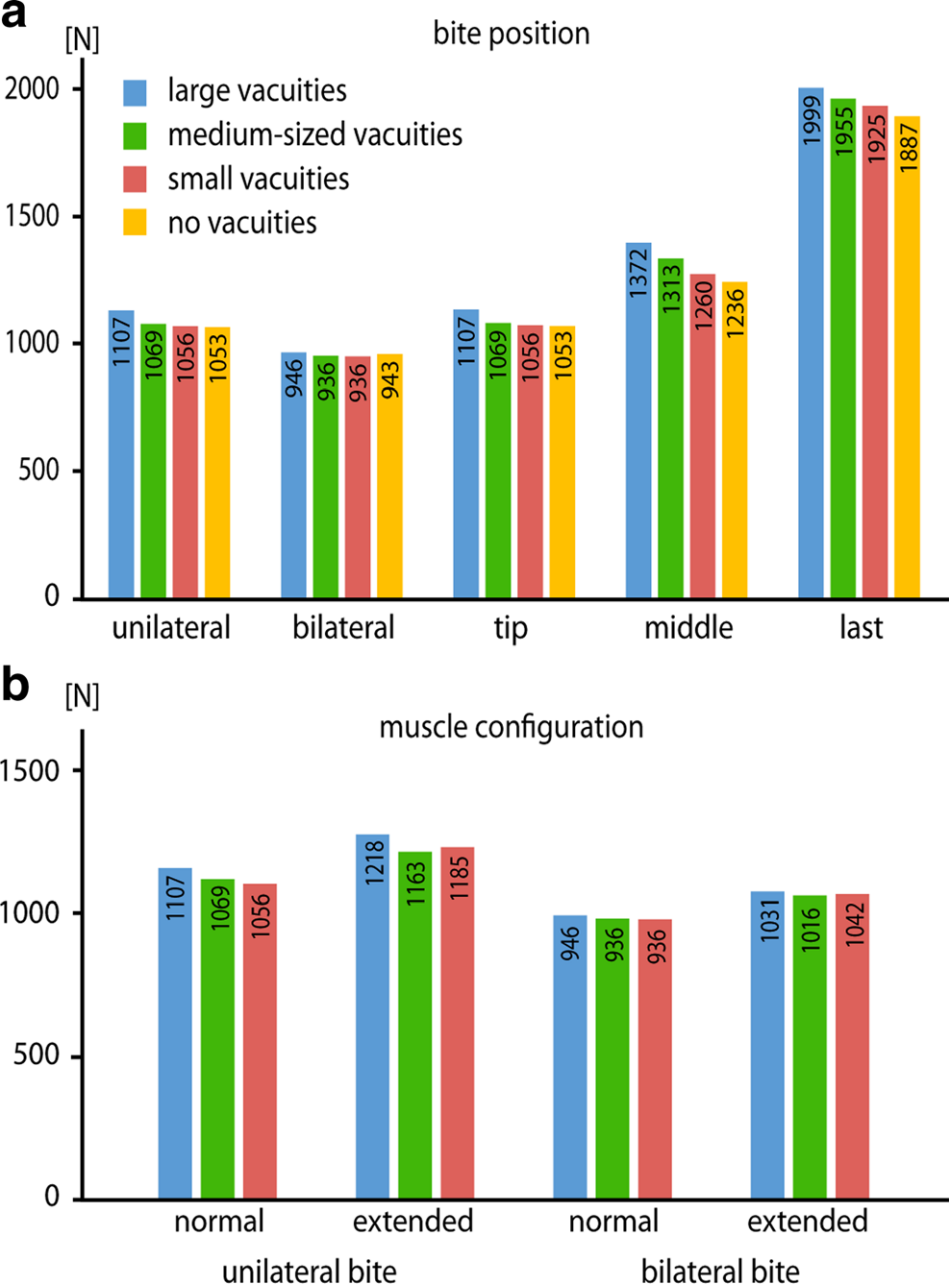
Bite forces obtained from the FEA models of tested configurations for **a** different bite positions and **b** different muscle configurations

Bite forces obtained from the finite element models showed that there are distinct differences between the tested palatal configurations. The original model with large interpterygoid vacuities recorded the highest bite forces for all simulated bite scenarios. Respectively, the hypothetical models with the closed interpterygoid vacuities consistently produced the lowest bite forces (Fig. 5). As was to be expected, bite forces increased with a posterior shift of the bite position, due to the skull acting as a third-class lever. Similarly, bite forces were higher for unilateral bite simulations than for bilateral biting.

An extension of the muscle attachments onto the margins of the interpterygoid vacuities resulted in distinctly higher bite forces for all tested configurations and unilateral and bilateral bite simulations.

## Discussion

Results of the finite element analyses demonstrated that palate morphology and the configuration of the interpterygoid vacuities have only minor effects on the cranial stability of the tested temnospondyl models. Stress and strain distribution patterns and magnitudes remained largely unchanged regardless of the size of the vacuities. These results indicate that the presence of vacuities and an increase in size did not lead to biomechanical disadvantages. In contrast, vacuity size showed a notable, positive correlation with bite force, suggesting that large interpterygoid vacuities are biomechanically more beneficial in transmitting muscle forces (Fig. 5a). In addition, the modeled extension of the m.AMIa onto the margins of the interpterygoid vacuities led to a similar effect in increasing bite forces in all tested scenarios. Due to the closed skull roof and adductor chamber, a dorsal expansion of the jaw closing musculature as found in most lissamphibians and amniotes did not evolve in temnospondyls (Säve-Söderbergh 1945; Carroll and Holmes 1980; Witzmann and Schoch 2013). Constrained in this way, the adductor musculature, and in particular the m.AMIa, expanded anteriorly and probably onto the margins of the interpterygoid vacuities. As indicated by the results of the finite element analyses, enlargement of the vacuities appears to offer the dual benefit of providing additional muscle attachment areas and allowing for more effective force transmission without compromising cranial stability. While not modeled in this study to allow for comparability of the different models, an extension of the muscle attachment sites would most likely be accompanied by an increase of muscle mass, thereby further increasing muscle and bite forces.

Although the results suggest that large interpterygoid vacuities provide biomechanical advantages, these results should be evaluated in the light of the modeled taxon. *P. helgolandicus* exhibits naturally large interpterygoid vacuities, whereas the other configurations had been created in a theoretical framework. Different morphological configurations of interpterygoid vacuities found in temnospondyls can correlate with different skull forms. Therefore, it cannot be ruled out that taxa with smaller interpterygoid vacuities possessed a functionally different cranial configuration, which counteracted the disadvantages. Basal tetrapods, such as *Acanthostega gunnari*, possessed a synovial joint between the palate and the braincase, which could have allowed some movement between elements and played a role in stress dissipation (Clack 2012; Porro et al. 2015). However, this mobility in articulation appears to have been reduced in temnospondyls as indicated in *Balanerpeton woodi* (Clack 2012), one of the first members of the group. Stress-mitigating effects of the palate-braincase joint are therefore likely to have been minimal, considering the evolution of a strong and akinetic suture in taxa more deeply nested in the phylogeny.

The neutral effects of interpterygoid vacuities on stress and strain distribution reflect results from previous studies on temnospondyl cranial biomechanics. Orbit size and position were found to play only a minor role in stress distribution and dissipation when tested in different, albeit simplified, cranial configurations of the temnospondyl *Edingerella madagascariensis* (Marcé-Nogué et al. 2015). Similarly, the functional analysis of different capitosaurian skull models indicated that the closure of the otic notch in “cyclotosaurs” did not affect the biomechanical behavior (Fortuny et al. 2012).

Throughout their evolutionary history, temnospondyls displayed a large variety of skull shapes, ranging from dorsoventrally flattened (platyrostral) and compact forms to long-snouted and mediolaterally narrow skulls (Angielczyk and Ruta 2012), providing evidence for the morphological diversity of the cranial skeleton among this group. The large disparity regarding cranial features, such as orbit and interpterygoid vacuity size, appears to reflect this overall pattern of cranial evolution. However, in comparison to other temnospondyl groups, capitosaurs (such as *P. helgolandicus*) displayed only limited cranial diversity and a conservative pattern of skull roof evolution (Stayton and Ruta 2006). It is generally assumed that capitosaurs filled the niche of large semiaquatic predators analogue to extant crocodilians (Milner 1990; Schoch and Milner 2000). It seems plausible that functional requirements, such as a biomechanically resistant skull adapted for powerful biting, considerably restricted morphological plasticity. Individual features within the cranial structure, such as the interpterygoid vacuity, however, permitted morphological variation without compromising overall stability.

Apart from functional considerations associated with stress distribution, muscle attachment or breathing, mass reduction effects of the interpterygoid vacuities could have been possible, in particular regarding the trend toward increased terrestriality in some temnospondyls (Reisz et al. 2009). However, calculations of the different cranial configurations showed only moderate mass-saving benefits (Table 2). Differences in mass between the fully closed palatal configuration and the model with large vacuities reached a maximum of 13 % (and considerably lower for the other configurations). Furthermore, a soft tissue membrane embedded with bony platelets most likely covered the interpterygoid vacuities in many temnospondyl taxa (Schoch 2013), thereby further reducing potential mass-saving effects. Such mass-saving benefits might have been more pronounced in and important for large taxa, in which the cranial skeleton increases with positive allometry. However, considering the results from this study, it is more likely that the evolution of large interpterygoid vacuities was linked to cranial stability, beneficial effects on bite force, and providing additional muscle attachments.

**Table 2:** Mass calculations of the different tested cranial configurations

Configuration	Mass (g)	Mass reduction (%)
No vacuities	4167	
Small vacuities	4562	2.65
Medium-sized vacuities	4689	5.29
Large vacuities	4817	13.49

Masses were calculated using specific density of bone (1770 kg/m^3^). Mass reduction ratios are calculated in respect to the model with no interpterygoid vacuities

## Conclusions

Our results demonstrate that the presence of large interpterygoid vacuities had only negligible effects on stress distribution and magnitudes in the tested temnospondyl cranial models. Rather, an increase in the size of interpterygoid vacuities concomitantly resulted in optimized muscle force transmission and increased bite forces and provided additional muscle attachment sites. Most likely these effects allowed for increased cranial plasticity in temnospondyl evolution. Mass-saving effects through the reduction of bone were found to be minimal and probably not the driving force behind morphological diversity in temnospondyl crania.

## Electronic supplementary material

Supplementary figure 1 Deformation contour plots for different tested cranial configurations. (PDF 274 kb)

Supplementary figure 2 Maximum principal stress contour plots for different tested cranial configurations. (PDF 317 kb)

Supplementary figure 3 Minimum principal stress contour plots for different tested cranial configurations. (PDF 324 kb)

Supplementary figure 4 Deformation contour plots for different tested cranial configurations and bite points. (PDF 163 kb)

Supplementary figure 5 Maximum principal stress contour plots for different tested cranial configurations and bite points. (PDF 180 kb)

Supplementary figure 6 Minimum principal stress contour plots for different tested cranial configurations and bite points. (PDF 182 kb)

Supplementary figure 7 Deformation magnitudes for different tested cranial configurations. (PDF 388 kb)

Supplementary figure 8 Maximum principal strain magnitudes for different tested cranial configurations. (PDF 465 kb)

Supplementary figure 9 Minimum principal strain magnitudes for different tested cranial configurations. (PDF 194 kb)

Supplementary figure 10 Von Mises stress, deformation and strain contour plots for different muscle configurations for models with no interpterygoid vacuities. (PDF 299 kb)
