# Supplementary material for: Palate anatomy and morphofunctional aspects of interpterygoid vacuities in temnospondyl cranial evolution
Source: Naturwissenschaften. 2016 Sep 14;103(9):79. doi: 10.1007/s00114-016-1402-z (PMC5023724; doi:10.1007/s00114-016-1402-z)
Supplement: Supplementary file 2 — Maximum principal stress contour plots for different tested cranial configurations. (PDF 317 kb) [file 114_2016_1402_MOESM2_ESM.pdf]

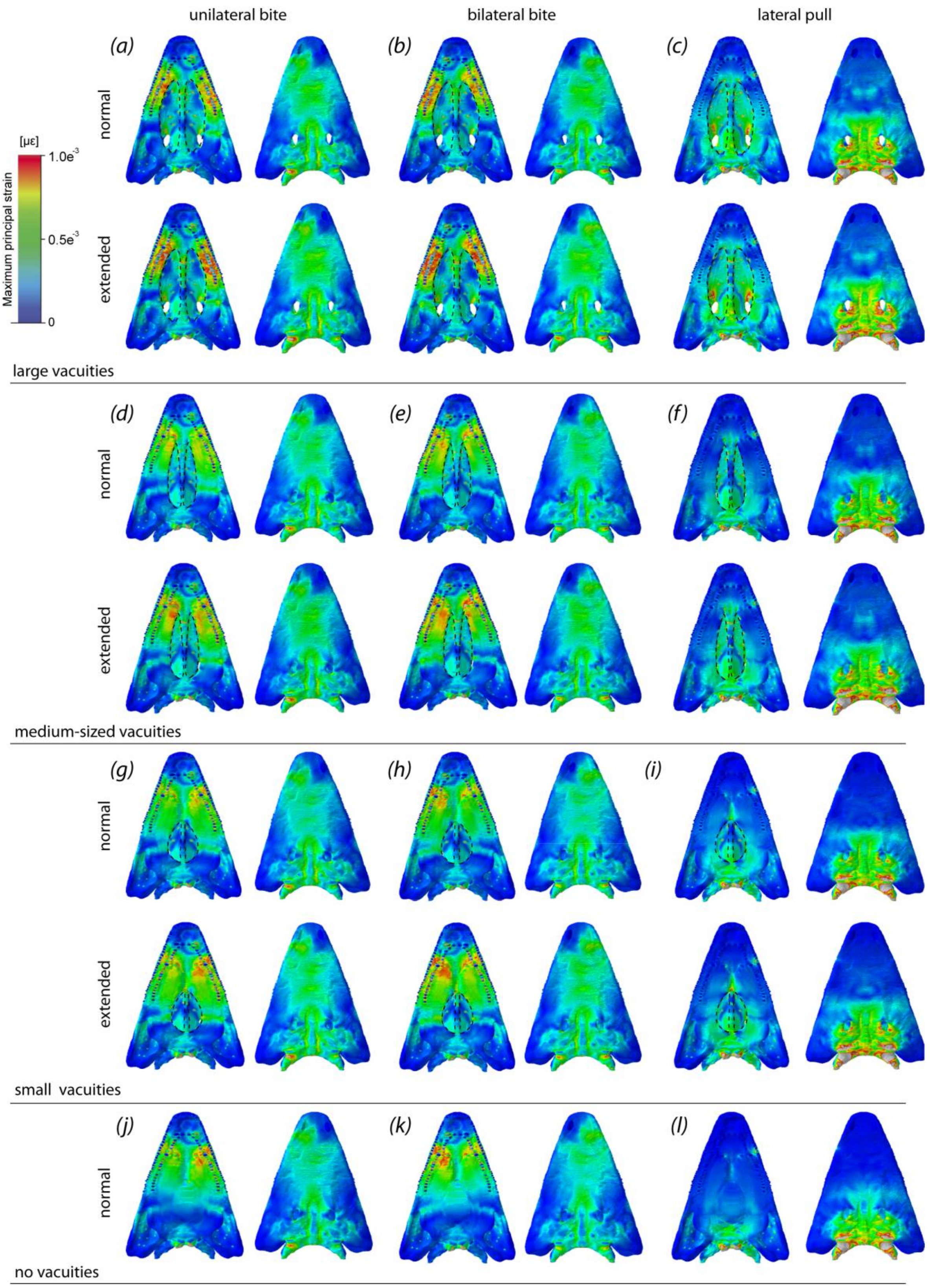

Supplementary figure 2 Maximum principal strain contour plots for different tested cranial configurations: (a-c) Original model, (d-f) medium-sized interpterygoid vacuities, (g-i) small interpterygoid vacuities, (j-l) closed palatal region. Different loading conditions: (a, d, g, j) unilateral bite on left side, (b, c, h, k) bilateral bite, (c, f, i, l) lateral pull to left side. Each in ventral and dorsal view. Location and size of the vacuities highlighted by stippled line.
