# Supplementary material for: Palate anatomy and morphofunctional aspects of interpterygoid vacuities in temnospondyl cranial evolution
Source: Naturwissenschaften. 2016 Sep 14;103(9):79. doi: 10.1007/s00114-016-1402-z (PMC5023724; doi:10.1007/s00114-016-1402-z)
Supplement: Supplementary file 5 — Maximum principal stress contour plots for different tested cranial configurations and bite points. (PDF 180 kb) [file 114_2016_1402_MOESM5_ESM.pdf]

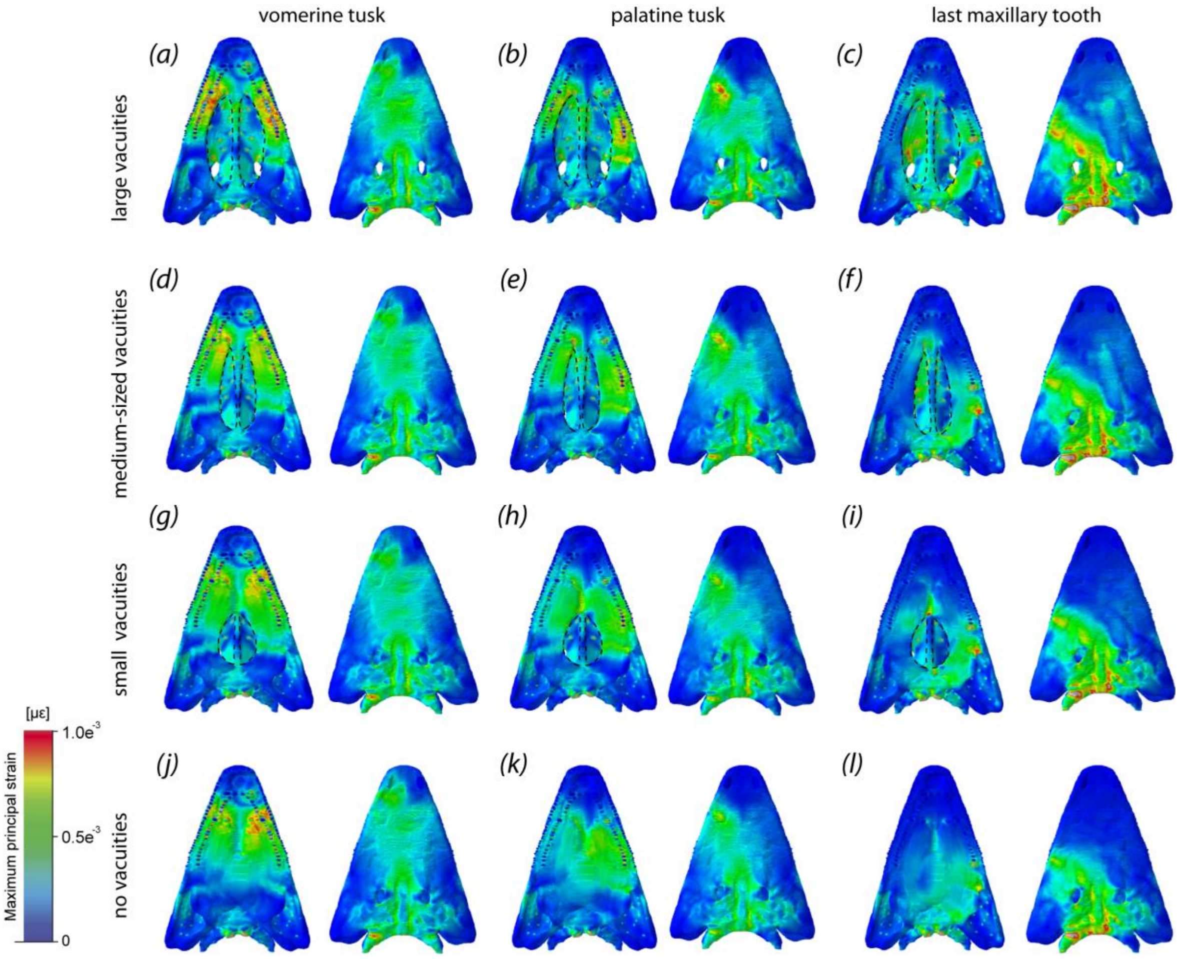

Supplementary figure 5 Maximum principal strain contour plots for different tested cranial configurations and bite points: (a-c) Original model, (d-f) medium-sized interpterygoid vacuities, (g-i) small interpterygoid vacuities, (j-l) closed palatal region. Different bite points: (a, d, g, j) vormerine tusk, (b, c, h, k) palatine tusk (c, f, i, l) last maxillary tooth. Each in ventral and dorsal view. Location and size of the vacuities highlighted by stippled line.
