# Supplementary material for: Palate anatomy and morphofunctional aspects of interpterygoid vacuities in temnospondyl cranial evolution
Source: Naturwissenschaften. 2016 Sep 14;103(9):79. doi: 10.1007/s00114-016-1402-z (PMC5023724; doi:10.1007/s00114-016-1402-z)
Supplement: Supplementary file 9 — Minimum principal strain magnitudes for different tested cranial configurations. (PDF 194 kb) [file 114_2016_1402_MOESM9_ESM.pdf]

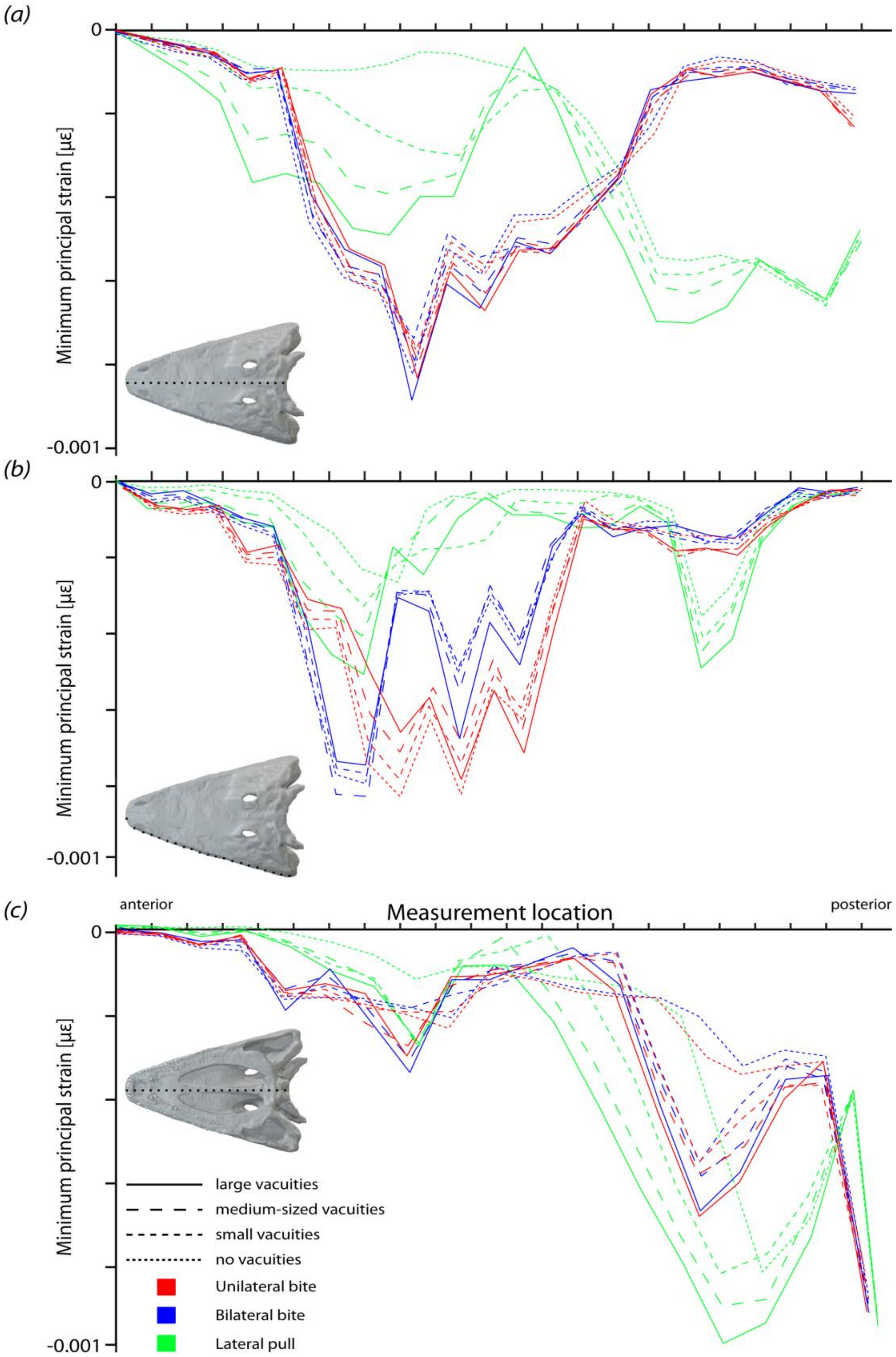

Supplementary figure 9 Minimum principal strain magnitudes for different tested cranial configurations. Measured along (a) the skull roof, (b) the left lateral margin, (c) the ventral midline.
