# Supplementary material for: Palate anatomy and morphofunctional aspects of interpterygoid vacuities in temnospondyl cranial evolution
Source: Naturwissenschaften. 2016 Sep 14;103(9):79. doi: 10.1007/s00114-016-1402-z (PMC5023724; doi:10.1007/s00114-016-1402-z)
Supplement: Supplementary file 10 — Von Mises stress, deformation and strain contour plots for different muscle configurations for models with no interpterygoid vacuities. (PDF 299 kb) [file 114_2016_1402_MOESM10_ESM.pdf]

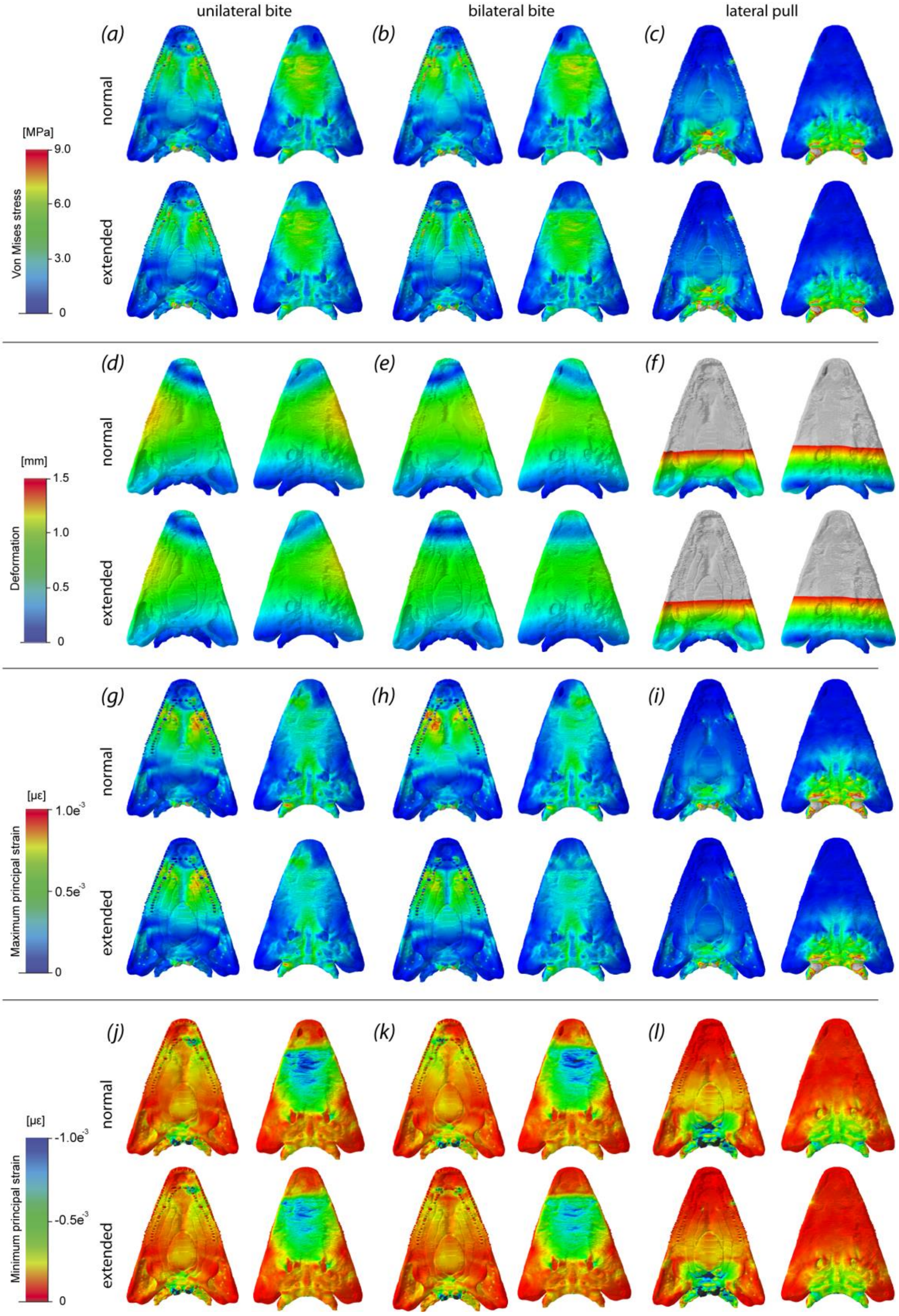

Supplementary figure 10 Contour plots for different muscle configurations for models with no interpterygoid vacuities. (a-c) Von Mises stress, (d-f) deformation, (g-i) maximum principal strain, and (j-l) minimum principal strain for normal and extended muscle attachments.
